# Patients with Moyamoya Vasculopathy Evaluated at a Single-Center in The Netherlands; Clinical Presentation and Outcome

**DOI:** 10.3390/jcm10091898

**Published:** 2021-04-27

**Authors:** Annick Kronenburg, Rachel Kleinloog, Albert van der Zwan, L. Jaap Kappelle, Luca Regli, Kees P. J. Braun, Catharina J. M. Klijn

**Affiliations:** 1 Department of Neurology and Neurosurgery, UMC Utrecht Brain Center, G03.129, P.O. Box 85500, 3508 GA Utrecht, The Netherlands; Rachelkleinloog@gmail.com (R.K.); A.vanderzwan@umcutrecht.nl (A.v.d.Z.); L.Kappelle@umcutrecht.nl (L.J.K.); K.Braun@umcutrecht.nl (K.P.J.B.); Karin.Klijn@radboudumc.nl (C.J.M.K.); 2 Department of Neurosurgery, Clinical Neuroscience Center, University Hospital Zurich, University of Zurich, 8091 Zurich, Switzerland; Luca.Regli@usz.ch; 3 Department of Neurology, Donders Institute for Brain, Cognition and Behavior, Center for Neuroscience, Radboud University Medical Center, 6500 HB Nijmegen, The Netherlands

**Keywords:** moyamoya, children, adults, Western world, functional outcome, follow-up, revascularization

## Abstract

Information on presentation and outcome of moyamoya vasculopathy (MMV) in European countries is limited. We investigated patient characteristics, treatment and outcome of patients with MMV. We retrieved patient characteristics and treatment information and determined functional outcome (modified Rankin Score (mRS); type of school/work) by structured telephone interviews. We performed uni- and multivariable logistic regression analysis to determine predictors of poor outcome. We included 64 patients with bilateral MMV. In children (31 patients), median age was 5 years (interquartile range (IQR) 2–11) and in adults (33 patients), it was 33 years (IQR 28–41). Predominant mode of presentation was ischemia (children 84%; adults 88%). Modified Rankin Scale (mRS) at presentation was ≤2 in 74%. Revascularization was performed in 42 patients (23 children). Median follow-up time was 46 months (IQR 26–90). During this period, 16 patients had recurrent stroke(s) and four patients died. In 73% of the patients (83% surgical group; 55% medically treated group), mRS was ≤2; 46% were able to return to regular school or work, of whom only 41% were on the same level. Univariable analysis revealed that surgical treatment was associated with lower odds of poor outcome ((mRS ≥ 3), OR 0.24; *p* = 0.017). This association was no longer statistically significant (OR 3.47; *p* = 0.067) in the multivariable model, including age and diagnosis (moyamoya disease or moyamoya syndrome). In this cohort of patients with MMV who presented in a single European center, a large proportion had good functional outcome. Nevertheless, less than half were able to attend regular school or were able to work at their previous level, indicating a large impact of the disease on their life.

## 1. Introduction

Moyamoya vasculopathy (MMV) is a cerebrovascular disorder of largely unknown etiology characterized by progressive stenosis or occlusion of the supraclinoid internal carotid arteries and their proximal branches [1,2]. Idiopathic MMV is referred to as moyamoya disease (MMD); patients in whom MMV is associated with another condition (e.g., Down syndrome or neurofibromatosis) are diagnosed as having moyamoya syndrome (MMS) [1]. Reported incidences of MMV vary between regions: from 0.086/100,000/year in the Western USA to around 1.0/100,000/year in Japan and Korea [3,4,5]. Familial cases are more frequent in Asia than in the Western world [5]. Patients typically present with transient ischemic attacks (TIAs) or ischemic stroke. Adults more often than children may also present with hemorrhagic stroke [3]. In addition, patients can present with headache, seizures and cognitive impairment [6]. Revascularization has been reported to reduce the risk of ischemic stroke, although randomized controls are lacking. Recently, revascularization surgery has been also suggested to prevent hemorrhages [7,8]. Revascularization surgery is generally recommended in patients with recurrent or progressive ischemic symptoms or affected cerebral blood flow or vascular reserve [9,10]. Information on long-term functional outcome and especially the ability to resume work or school is limited. In this study, we assessed clinical features, treatment and outcome of patients with MMV referred to a single neurosurgical expertise center in the Netherlands.

## 2. Methods

### 2.1. Patient Selection

We identified consecutive patients with bilateral MMV referred to the University Medical Center (UMCU) between January 1986 and October 2012 (the start of a prospective MMV cognition study). The diagnosis of MMV was confirmed by digital subtraction angiogram or magnetic resonance angiography according to the criteria of the Research Committee on Spontaneous Occlusion of the Circle of Willis (Moyamoya Disease) in Japan [9]. The data that support the findings of this study are available from the corresponding author upon reasonable request.

### 2.2. Retrospective Chart Review

All charts were reviewed for the following data: sex; age at onset; type of first and recurrent symptoms: acute ischemic stroke (AIS), (recurrent) TIAs (defined as symptoms <24 h without permanent deficits or new ischemic brain lesion), slowly progressive neurological symptoms with infarction on imaging, hemorrhage, seizures, headache, or no symptoms; time passed (interval) from first symptom until presentation to a neurosurgical center; modified Rankin Scale [11] (mRS, in patients >4 years old) at time of presentation to a neurosurgical center; associated disorders; diagnostic imaging performed; medical treatment; side and type of neurosurgical revascularization (combined; direct; or indirect); major complications of treatment (within 30 days: AIS, epi-/subdural or intracerebral hematoma and whether re-operation was performed); and recurrent symptoms before and after treatment.

### 2.3. Follow-Up Telephone Interview

Patients or their parents/legal guardians were interviewed by phone (by A.K. and R.K.) to retrieve data on: ethnicity; family history of MMV; recurrent symptoms; current medical therapy; and mRS at time of follow up [12,13]. Good outcome was defined as mRS ≤ 2. We asked patients whether they were able to work or attend regular school and whether they functioned at the same level as before their first symptoms.

### 2.4. Data Analysis

We analyzed differences in patient characteristics between patients with childhood (<18 years old) and adult onset and differences between patients who underwent surgical therapy and those who received medical treatment alone by using descriptive statistics. To determine possible predictors for good outcome (mRS ≤ 2), recurrent stroke, and functioning at the previous level (according to the type of school or work), we performed uni- and multivariable logistic regression analysis with predefined clinically relevant predictors.

## 3. Results

### 3.1. Baseline Characteristics

**Figure 1:**
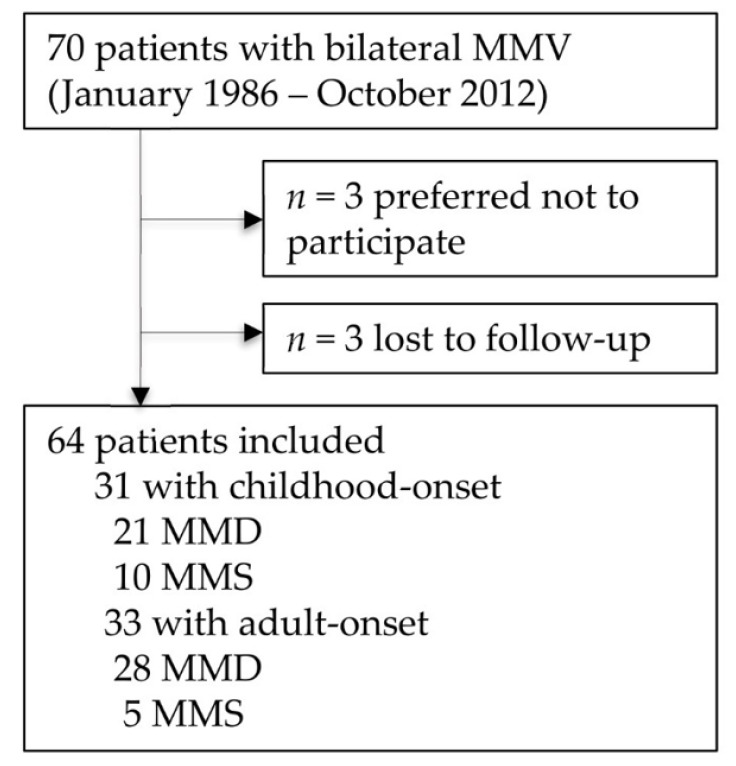
Flowchart patient selection. MMD = moyamoya disease; MMS = moyamoya syndrome; MMV = moyamoya vasculopathy.

We identified 70 patients with bilateral MMV (Figure 1) of whom we included 64. Three patients preferred not to participate, another 3 were lost to follow-up. Of 64 patients, 31 (48%) presented in childhood (Table 1). Median age of onset in children was 5 years (inter quartile range (IQR 2–11) and in adults 33 years (IQR 28–41). Fifteen (23%) were diagnosed with MMS (Supplementary Table S1). The female to male ratio was 2.6:1, with a higher percentage of females in adults than in children (Table 1). Forty-two patients (66%) were of Caucasian descent (Supplementary Table S2). One Caucasian patient had a positive family history of MMV with the sister of the mother affected. The majority of patients presented with ischemic symptoms, both in adults and in children (Table 1).

**Figure 2:**
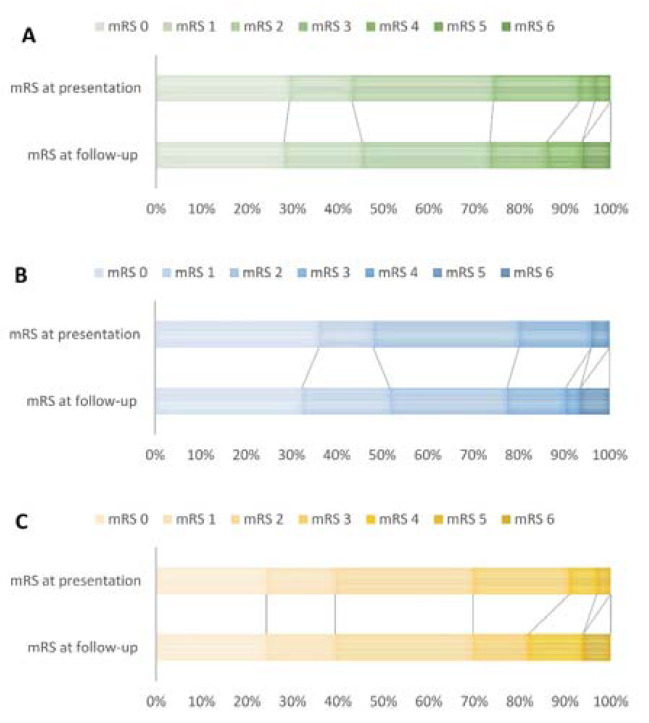
Distribution of the proportions of modified Rankin Scales (mRS) for: (**A**) complete group (missing n = 6 at presentation); (**B**) with childhood onset (missing n = 6 at presentation); (**C**) with adult onset.

The median interval from first symptoms to referral to a neurosurgical center was 8 months (IQR 2–45), 6 months in children (IQR 2–55) and 16 months in adults (IQR 5–45; Table 2). Before referral, recurrent ischemic or hemorrhagic stroke(s) had occurred in 29 patients (45%, 11 children and 18 adults). At the time of presentation, 43 patients (74%) had a mRS of ≤2 (20 children, 80%; 23 adults, 70%; Figure 2).

### 3.2. Treatment and Treatment Outcome

Forty-two patients were treated surgically and 22 received medical treatment only. The median interval between first symptoms and referral was 7 months (IQR 3–41) in the surgical group and 25 months (IQR 2–64) in the medically treated patients.

The decision to operate or not was made by a multidisciplinary team of neurologists and neurosurgeons. The main reasons not to perform surgery were: the absence of clinical symptoms after presentation (n = 14), patients who presented with intracerebral hemorrhage (n = 4), individual patient-related risks of surgery (n = 1), and fibromuscular dysplasia (FMD) with multi-organ involvement and extensive neurological injury at presentation (n = 1).

#### 3.2.1. Surgical Group

**Figure 3:**
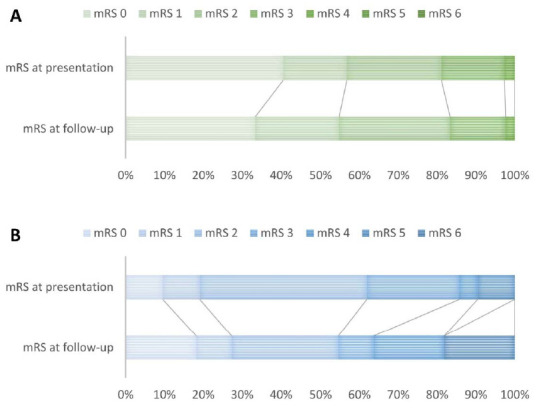
Distribution of the proportions of modified Rankin Scales (mRS) for (**A**) surgically treated group (missing n = 5 at presentation); (**B**) medically treated group (missing n = 1 at presentation).

**Figure 4:**
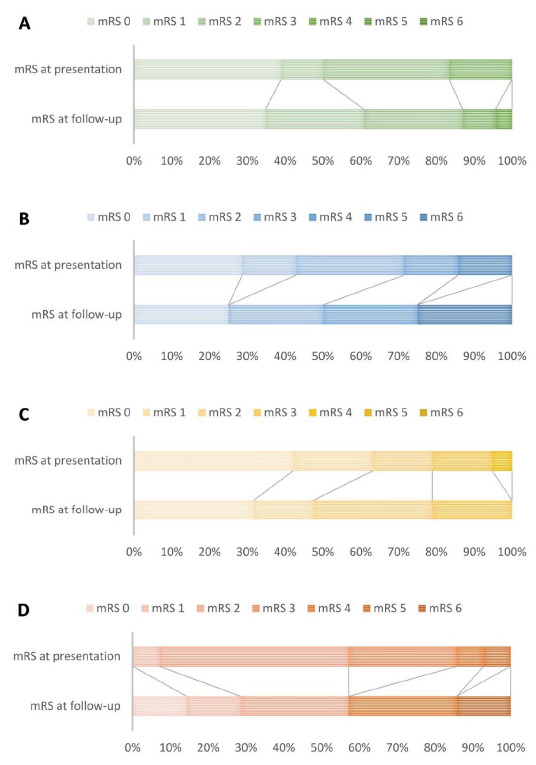
Distribution of the proportions of modified Rankin Scales (mRS) for: (**A**) surgically treated group with childhood-onset (missing n = 5 at presentation); (**B**) medically treated group with childhood-onset (missing n = 1 at presentation); (**C**) surgically treated group with adult-onset; (**D**) medically treated group with adult-onset.

Of the 42 surgically treated patients, 35 were treated in the UMCU, and 7 were treated elsewhere (one in the Medical Center Haaglanden (1997), one in the Children’s Hospital Boston, Harvard Medical School, and 5 in the University Hospital of Zürich, Switzerland [14] (period 2001–2005)). One patient treated in the UMCU in 2009 (direct bypass) had been operated before elsewhere (1990, indirect frontal bypass). Thirty of the 42 patients (81%) had a mRS ≤ 2 at presentation (Figure 3A and Figure 4A,C). The median time between presentation to a neurosurgical center and the first surgical procedure was 2 months (IQR 1–8). Twenty-one of the 42 patients were treated bilaterally (50%; 19 children). Twenty-nine patients underwent revascularization of the middle cerebral artery (MCA) territory only, by either a direct superficial temporal artery (STA)-MCA bypass, an indirect procedure (encephaloduro(arterio)myosynangiosis; 41 hemispheres), or a combined procedure. In 10 patients, in addition to bilateral MCA revascularization, revascularization of the frontal territory was performed; either by a direct STA-anterior cerebral artery bypass or indirect procedures (encephaloduroperisteomyosynangiosis (EDPS) [15], arteriosynangiosis or burr holes). In three patients, bilateral EDPS was performed after revascularization of a single MCA territory. Acetylsalicylic acid was prescribed in all patients but one who had had an hemorrhagic stroke.

#### 3.2.2. Perioperative Events

In two patients, surgery was complicated by an AIS. In a 4-year-old child, an AIS occurred directly after surgery in the operated hemisphere with worsening of pre-existing motoric dysphasia and a new distal right-arm paresis (mRS from 1 pre-operatively to 2 at discharge). At latest follow-up, after 14 months, the child had a mRS of 1 with only mild coordination deficits of the right-hand. In a 14-month-old child, AIS occurred postoperatively in the non-operated hemisphere resulting in a unfavorable long-term outcome with a severe persistent hemiparesis and cortical ischemia and atrophy on MRI. Two other children had a sub- or epidural hematoma that required surgical evacuation without a worsening of mRS at discharge and a favorable outcome with a mRS of 1 and 0 at follow-up without new persistent neurological deficits.

#### 3.2.3. Medically Treated Group

Thirteen of 21 medically treated patients (62%, 5 children and 8 adults) had a mRS ≤ 2 at presentation to a neurosurgical center. Antiplatelet therapy was prescribed in 16 of 22 medically treated patients (73%; six children (75%) and 10 adults (71%)). Antiplatelets were not prescribed in five patients with a history of hemorrhage stroke and in one patient with severe comorbidities.

### 3.3. Long Term Outcome

Median follow-up time between first presentation to a neurosurgical center and follow up was 46 months (IQR 26–90). At follow-up, 90% of patients used antiplatelet therapy; Table 2). Eighteen patients had improvement of mRS (31%, eight with childhood onset); 17 deteriorated (29%, six with childhood onset); and 23 remained stable (40%, 11 with childhood-onset, Supplementary Table S3).

#### 3.3.1. Surgical Group

During the follow-up period (median 41 months, IQR 23–92), 10 patients (24%, 4 children) had a recurrent stroke, of these three (all adults) occurred after surgery and two peri-operatively.

In one patient, a recurrent AIS occurred after initial medical treatment only (mRS from 0 to 1, no new neurological deficits), and revascularization was subsequently performed. Four patients had a recurrent stroke pre-operatively: one adult during the diagnostic work-up for a suspected intracranial vasculopathy after radiotherapy (stable mRS and no new permanent deficits at neurological examination); one adult who had a hemorrhagic stroke one month after presentation to the neurosurgical center (mRS 0 at that time), which resulted in a mRS of 4; two children during the pre-operative work-up, resulting in an increase in mRS from 0 to 1 (no new neurological deficits) in one child and the other had recurrent asymptomatic AIS during a fever. After revascularization, three adults had a recurrent stroke in the treated hemisphere 4, 10, and 25 months after surgery. Neurological deficits were mild, and the bypass was patent on imaging studies in all patients; mRS remained stable in two patients (mRS 2 and 3) and worsened in one patient from score 1 to 2.

At the time of the last follow up, 35 of 42 surgically treated patients (83%) had a mRS ≤ 2 at follow-up (20 with childhood (87%) and 15 with adult onset (79%)). Eleven had an improved mRS (30%, six with childhood onset), 10 had deteriorated (27%, four with childhood onset); and 16 remained stable (43%, eight with childhood onset; in five children, no baseline mRS was available (<age 4)). Eighteen patients (50% (five children under age 4; missing data in one adult) were able to function on the same level as before the first symptoms (11 with childhood (61%) and seven with adult onset (39%). Eleven patients with childhood onset (50%) were able to go to a regular school or work; seven with adult onset (39%) were able to work.

#### 3.3.2. Medically Treated Group

Eight of 22 patients (36%, two children, Table 2) had one or more recurrent strokes during a median follow-up time of 61 months (IQR 34–89) from first presentation to a neurosurgical center and follow-up. One patient had recurrent strokes before MMV diagnosis. One child had a recurrent intraventricular hemorrhage with an increase in mRS from 2 to 3. Two children had multiple AIS, which at follow-up resulted in persistent tetraparesis, dysphasia and intellectual disability (mRS 5). An adult had a recurrent hemorrhagic stroke with an increase of his mRS from 2 to 3, which recovered with mildly persistent dysphasia (mRS 2). Two adults had AIS 2 days and 9 months after presentation to a neurosurgical center and no new neurological deficits with a stable mRS of 2. Another patient had multiple AIS 3 years after presentation, which resulted in a persistent increase in mRS from 3 to 4 with hemianopia at follow-up.

Four patients (two children) died 0, 16, 41 and 57 months after presentation to the neurosurgical center. Both children had MMS related to FMD, with systemic vasculopathy and multi-organ involvement [16]. One died of pulmonary bleeding as a complication of balloon dilatation of stenosis of the pulmonary artery. The other child had a sudden death as a result of multiple infarctions in heart and lungs due to FMD [16]. One adult died because of two hemorrhagic strokes during the diagnostic workup. Another adult patient was treated conservatively after an AIS without recurrent symptoms and died due to pneumonia.

In the medically treated group, 12 of 22 patients (55%) had a mRS ≤ 2 at follow up (four with childhood (50%) and eight with adult onset (57%); Figure 3B and Figure 4B,D). Seven patients had improvement in mRS (33%, two with childhood-onset); seven deteriorated (33%, two with childhood-onset); and seven remained stable (33%, three with childhood-onset). Only three patients (20%; two with childhood (50%) and one with adult onset (9%)) were able to function at the same level as before the first symptom of MMV. Eight adults (47%, of whom three with childhood onset) were able to work, whereas none of the children attended a regular school.

### 3.4. Determinants of Outcome

Univariable analysis (Supplementary Table S4) showed that surgical treatment was associated with lower odds of poor outcome ((mRS ≥ 3), OR 0.24, 95% CI 0.075–0.771, *p* = 0.017) and had a trend towards lower odds for recurrent stroke (OR 0.31, 95% CI 0.099–0.998; *p* = 0.050). These associations were no longer significant in the multivariable analysis (Table 3; OR 3.47, 95% CI 0.914–13.135; *p* = 0.067 and OR 2.98, 95% CI 0.847–10.459; *p* = 0.089 respectively).

## 4. Discussion

In this single center cohort in the Netherlands, adults and children with MMV predominantly presented with symptoms of ischemia. In patients selected for surgery, 73% had a stable or improved mRS over time and 83% eventually had a mRS ≤ 2. Nevertheless, less than half of the surgically treated patients could attend regular school or work, indicating a significant impact of the disease on the quality of life despite treatment. In the medically treated group, 55% had a mRS ≤ 2 and only 20% could function on the same level as before their first symptoms, and less than half could attend regular school or work.

When compared to Asian cohorts, a Western moyamoya phenotype has been characterized by a more pronounced female preponderance, higher than average age at onset of the disease, relative lack of family history and lower chance of hemorrhagic stroke [4]. The characteristics of the patients in our cohort are in line with this description.

The high risk of recurrent stroke in medically treated patients in our study is comparable to the annual rate of recurrent ischemic stroke of 13.3% in an untreated adult North-American population [17] and a 5-year risk of recurrent stroke of 81% in a German cohort [18]. In our cohort, 19% of the surgically treated patients had a recurrent stroke during a median time of follow up of 41 (IQR 23–92) months, of which 3 of 42 (7%) occurred after the perioperative period, which is in line with 5-year event rates of 5.5 and 5.8% in North-American cohorts [8,19].

The number of patients with a favorable mRS at baseline and at follow-up was lower in the medically treated group (55%) compared to the surgical group (83%). Obviously, selection bias limits this observation, because selection for surgery has been less likely for patients in a poor clinical condition. Several studies have shown improvement of preoperative mRS in surgically treated patients, with an improvement of 1–2 points in 67.8% of the cases [8,20]. Despite our finding that the mRS in the surgical and medically treated group improved or remained stable in the large majority of patients, less than half of all patients were able to attend regular school or were able to work at their previous level. This is in accordance with a recent study, which demonstrated poor outcome in half of the children, with AIS as a significant predictor [21]. Given the observation that around 30% of patients with MMV have cognitive impairments [6], these results imply that cognitive disturbances may play an important role in the limitations to resume previous activities.

In our cohort, children seemed to have fewer recurrent strokes and a more favorable functional outcome than adults, as has been described by others [22,23]. One explanation could be that children are referred for surgical treatment faster than adults. In addition, children may have better brain plasticity than adults [24].

Although the effect of revascularization surgery has never been confirmed in a randomized clinical trial, surgery is thought to improve outcome in patients with MMV [8,9,10,25,26]. From the medically treated patients in this study, we learned that some patients may remain stable without the need for surgical intervention. Unfortunately, we could not identify predictors for a good outcome. We observed recurrent symptoms in eight patients in whom medical treatment was chosen, even after a period of more than 2 years without symptoms. Although this resulted in a shift from good to poor clinical outcome in only three patients, multiple observational studies have found that MMV is a progressive disease with an up to 80% risk of recurrent ischemic strokes in 5 years [18,27,28]. Other retrospective hospital-based studies suggested that recurrent strokes in MMD are most prevalent in the first 1–2 years after onset [18,27,28]. There is supportive evidence that revascularization surgery can prevent recurrent stroke and decline of both neurological and cognitive function [29,30]. Early referral of patients with MMV to a specialized center is therefore essential for timely consideration of surgical intervention [10,25]. Because surgical treatment is not without risk, the indication for, and timing of revascularization surgery should be decided in a multidisciplinary team, taking into account the individual patients characteristics, hemodynamic studies, and technical considerations regarding surgery [9,10]. In our cohort, we had a complication risk of 9.5%, which is within the range of other studies [22,26].

The strenghts of our study are the relatively large cohort for this rare disease and that we were able to investigate outcome at the level of work and school. Furthermore, there are very few studies on functional outcome in Western MMV. Our study also has limitations. First, our study was a retrospective analysis of a cohort referred over a long period of time. The approach of patients with MMV has changed over the years, including the selection criteria for surgery [10]. Second, although it would be preferable to determine clinical outcome in children by using the pediatric stroke outcome measure (PSOM), the retrospective character of this study only allowed us to determine the mRS combined with school type. However, school type is an important measure of cognitive outcome, and corresponds better with the doctor’s impression of outcome [13]. Last but not least confounding by indication hampers a reliable comparison between the outcomes after medical or surgical treatment.

## 5. Summary

In this Dutch cohort of patients with MMV, patients predominantly presented with ischemia. Although clinical outcome improved or remained stable in most patients, less than half of the patients were able to attend regular school or work at their previous level, indicating a large impact of the disease on their life. Early diagnosis and treatment might improve outcome and therefore early referral of patients to a center with the necessary neurovascular and neurosurgical expertise is essential.

## Figures and Tables

**Table 1:** Baseline characteristics of 64 patients with moyamoya vasculopathy.

		All	Surgical Group	Medical Group
Total number of patients (%)	Total group	64 (100)	42 (66)	22 (34)
	Children	31 (48)	23 (74)	8 (26)
	Adults	33 (52)	19 (58)	14 (42)
MMS, n (%)	Total group	15 (23)	9 (21)	6 (27)
	Children	10 (32)	5 (22)	5 (63)
	Adults	5 (15)	4 (21)	1 (7)
Median age at onset (years, IQR)	Total group	18 (5–33)	14 (4–31)	30 (11–42)
	Children	5 (2–11)	4 (2–8)	8 (2–11)
	Adults	33 (28–41)	32 (25–39)	35 (30–44)
Female sex, n (%)	Total group	46 (72)	31 (74)	15 (68)
	Children	17 (55)	13 (57)	4 (50)
	Adults	29 (88)	18 (95)	11 (79)
First presenting symptom n (%)				
Ischemia	Total group	55 (86)	38 (90)	17 (77)
	Children	26 (84)	20 (87)	6 (75)
	Adults	29 (88)	18 (95)	11 (79)
AIS	Total group	25 (39)	16 (38)	9 (41)
	Children	14 (45)	11 (48)	3 (38)
	Adults	11 (33)	5 (26)	6 (43)
TIA(s)	Total group	22 (34)	19 (45)	3 (14)
	Children	9 (29)	8 (35)	1 (13)
	Adults	13 (39)	11 (58)	2 (14)
Slowly progressive symptoms with infarction	Total group	8 (13)	3 (7)	5 (23)
	Children	3 (10)	1 (4)	2 (25)
	Adults	5 (15)	2 (11)	3 (21)
Hemorrhage	Total group	5 (8)	1 (2)	4 (18)
	Children	1 (3)	0	1 (13)
	Adults	4 (12)	1 (5)	3 (21)
Headache	Total group	2 (3)	1 (2)	1 (5)
	Children	2 (6)	1 (4)	1 (13)
	Adults	0	0	0
Seizures	Total group	2 (3)	2 (5)	0
	Children	2 (6)	2 (9)	0
	Adults	0	0	0

AIS = acute ischemic stroke; IQR = interquartile range; n = number of patients; TIA = transient ischemic attack.

**Table 2:** Outcome of 64 patients with moyamoya vasculopathy.

		All	Surgical Group	Medical Group
Median interval first symptoms presentation NSC (months, IQR)	Total group	8 (2–45)	7 (3–41)	25 (2–64)
	Children	6 (2–55)	6 (2–32)	73 (2–154)
	Adults	16 (5–45)	16 (7–45)	16 (2–34)
One or more recurrent ischemic/hemorrhagic stroke(s) before presentation NSC, n (%)	Total group	29 (45)	19 (45)	10 (45)
	Children	11 (35)	8 (35)	3 (38)
	Adults	18 (55)	11 (58)	7 (50)
mRS ≤ 2 at presentation NSC, n (%)	Total group	43 (74) *	30 (81)	13 (62)
	Children	20 (80)	15 (83)	5 (71)
	Adults	23 (70)	15 (79)	8 (57)
Treatment and recurrent events				
Median interval first contact NSC to first surgery (months, IQR)	Total group	-	2 (1–8)	-
	Children	-	3 (1–9)	-
	Adults	-	2 (1–4)	-
Median follow-up time after presentation NSC and telephone interview (months, IQR)	Total group	46 (26–90)	41 (23–92)	61 (34–89)
	Children	45 (17–95)	45 (17–95)	45 (19–147)
	Adults	55 (28–86)	37 (25–91)	66 (52–84)
Antiplatelets, n (%)	Total group	54 (90) ^†^	40 (95)	14 (78)
	Children	28 (97)	23 (100)	5 (83)
	Adults	26 (84)	17 (89)	9 (75)
One or more recurrent ischemic/hemorrhagic stroke(s) after presentation NSC (excluding operative complications), n (%)	Total group	16 (25) ^‡^	8 (19)	8 (36)
	Children	4 (13)	2 (9)	2 (25)
	Adults	12 (36)	6 (32) ^§^	6 (43)
Case fatality, n (%)	Total group	4 (6)	0	4 (18)
	Children	2 (6)	0	2 (25)
	Adults	2 (6)	0	2 (14)
Long-term outcome at time of telephone interview				
mRS ≤ 2, n (%)	Total group	47 (73)	35 (83)	12 (55)
	Childhood-onset ^| |^	24 (77)	20 (87)	4 (50)
	Adult-onset	23 (70)	15 (79)	8 (57)
Same level as before first symptoms, n (%)	Total group	21 (41) ^#^	18 (50)	3 (20)
	Childhood-onset ^| |^	13 (59)	11 (61)	2 (50)
	Adult-onset	8 (28)	7 (39)	1 (9)
Regular school/work, n (%)	Total group	26 (46) **	18 (45)	8 (47)
	Childhood-onset ^| |^	14 (50)	11 (50)	3 (50)
	Adult-onset	12 (41)	7 (39)	5 (45)

IQR = interquartile range; n = number of patients; mRS = modified Rankin Scale; NSC = neurosurgical center. * Of 6 children (5 surgical and 1 medically treated group), no mRS scores were available (<age 4); ^†^ 4 patients died (2 children and 2 adults in the non-surgery group); ^‡^ data of two medically treated patients with childhood onset is missing (in 1 child symptoms suspected for ischemia were reported during follow-up interview; in another child recurrent ischemic events were suspected clinically, but no additional imaging studies were performed due to the pre-existent poor condition of the child); ^§^ 3 adults had a recurrent stroke in the operated hemisphere; ^| |^ 9 patients had childhood-onset MMV and were adults at follow-up; ^#^ data of 13 patients are missing (4 patients died; 6 children were <4 years old at presentation or follow-up and not yet attending school; in 1 child with slowly progressive symptoms the onset of the disease could not be correlated to the level of functioning at that time; in 1 adult, data on return to work was not available; 1 adult was already retired after the symptomatic onset of moyamoya); ** data on 7 patients is missing (4 patients died; 1 adult retired; in 1 adult data on return to work was not available; 1 child not yet attending school).

**Table 3:** Multivariable logistic regression analysis.

Predictors	Poor Outcome (mRS ≥ 3)		Recurrent Stroke		No Normal School/Adult Does Not Work	
	Odds Ratio (95% CI)	*p* Value	Odds Ratio (95% CI)	*p* Value	Odds Ratio (95% CI)	*p* Value
Age of onset MMV	0.993 (0.952–1.037)	0.764	1.017 (0.977–1.059)	0.419	1.006 (0.965–1.049)	0.772
Diagnosis (MMD or MMS)	0.982 (0.173–5.585)	0.983	1.345 (0.302–5.980)	0.697	2.121 (0.497–9.055)	0.310
mRS presentation ≥ 3	*4.406 (0.984–19.728)*	*0.053*	0.827 (0.183–3.739)	0.805	3.679 (0.773–17.523)	0.102
# events (ICH/infarction) before presentation NSC	1.479 (0.730–2.995)	0.277	0.749 (0.364–1.541)	0.432	1.747 (0.888–3.434)	0.106
Surgically treated	*3.466 (0.914–13.135)*	*0.067*	*2.976 (0.847–10.459)*	*0.089*	0.728 (0.200–2.646)	0.629

CI = confidence interval; ICH: intracerebral hematoma; MMD = moyamoya disease; MMS = moyamoya syndrome; MMV = moyamoya vasculopathy; mRS = modified Rankin Scale; NSC = neurosurgical center; # = number.

## Data Availability

The data that support the findings of this study are available from the corresponding author upon reasonable request.

## Supplementary Materials

The following are available online at https://www.mdpi.com/article/10.3390/jcm10091898/s1, Table S1: Associated disorders, Table S2: Ethnicity or country of origin, Table S3: Modified Rankin scale at follow-up, Table S4: Univariable logistic regression analysis.

## References

[1] Scott R.M., Smith E.R. (2009). Moyamoya disease and moyamoya syndrome. N. Engl. J. Med..

[2] Kronenburg A., Braun K.P.J., van der Zwan A., Klijn C.J.M. (2014). Recent advances in moyamoya disease: Pathophysiology and treatment. Curr. Neurol. Neurosci. Rep..

[3] Kleinloog R., Regli L., Rinkel G.J.E., Klijn C.J.M. (2012). Regional differences in incidence and patient characteristics of moyamoya disease: A systematic review. J. Neurol. Neurosurg. Psychiatry.

[4] Hever P., Alamri A., Tolias C. (2015). Moyamoya angiopathy—Is there a Western phenotype?. Br. J. Neurosurg..

[5] Kim J.S. (2016). Moyamoya Disease: Epidemiology, Clinical Features, and Diagnosis. J. Stroke.

[6] Kronenburg A., van den Berg E., van Schooneveld M.M., Braun K.P.J., Calviere L., van der Zwan A., Klijn C.J. (2018). Cognitive Functions in Children and Adults with Moyamoya Vasculopathy: A Systematic Review and Meta-Analysis. J. Stroke.

[7] Miyamoto S., Yoshimoto T., Hashimoto N., Okada Y., Tsuji I., Tominaga T., Nakagawara J., Takahashi J.C., Yamada K., Tomata Y. (2014). Effects of extracranial-intracranial bypass for patients with hemorrhagic moyamoya disease: Results of the Japan Adult Moyamoya Trial. Stroke.

[8] Guzman R., Lee M., Achrol A., Bell-Stephens T., Kelly M., Do H.M., Marks M.P., Steinberg G.K. (2009). Clinical outcome after 450 revascularization procedures for moyamoya disease. Clinical article. J. Neurosurg..

[9] Research Committee on the Pathology and Treatment of Spontaneous Occlusion of the Circle of Willis, Health Labour Sciences Research Grant for Research on Measures for Infractable Diseases (2012). Guidelines for diagnosis and treatment of moyamoya disease (spontaneous occlusion of the circle of Willis). Neurol. Med. Chir..

[10] Ferriero D.M., Fullerton H.J., Bernard T.J., Billinghurst L., Daniels S.R., DeBaun M.R., deVeber G., Ichord R.N., Jordan L.C., Massicotte P. (2019). Management of Stroke in Neonates and Children: A Scientific Statement From the American Heart Association/American Stroke Association. Stroke.

[11] van Swieten J.C., Koudstaal P.J., Visser M.C., Schouten H.J., van Gijn J. (1988). Interobserver agreement for the assessment of handicap in stroke patients. Stroke.

[12] Janssen P.M., Visser N.A., Dorhout Mees S.M., Klijn C.J.M., Algra A., Rinkel G.J.E. (2010). Comparison of telephone and face-to-face assessment of the modified Rankin Scale. Cerebrovasc. Dis..

[13] Bulder M.M.M., Hellmann P.M., van Nieuwenhuizen O., Kappelle L.J., Klijn C.J.M., Braun K.P.J. (2011). Measuring outcome after arterial ischemic stroke in childhood with two different instruments. Cerebrovasc. Dis..

[14] Khan N., Yonekawa Y. (2008). Moyamoya angiopathy in Europe: The beginnings in Zurich, practical lessons learned, increasing awareness and future perspectives. Acta Neurochir. Suppl..

[15] Kronenburg A., Esposito G., Fierstra J., Braun K.P., Regli L. (2014). Combined Bypass Technique for Contemporary Revascularization of Unilateral MCA and Bilateral Frontal Territories in Moyamoya Vasculopathy. Acta Neurochir. Suppl..

[16] de Vries R.R., Nikkels P.G.J., van der Laag J., Broere G., Braun K.P.J. (2003). Moyamoya and extracranial vascular involvement: Fibromuscular dysplasia? A report of two children. Neuropediatrics.

[17] Gross B.A., Du R. (2013). The natural history of moyamoya in a North American adult cohort. J. Clin. Neurosci..

[18] Kraemer M., Heienbrok W., Berlit P. (2008). Moyamoya disease in Europeans. Stroke.

[19] Mallory G.W., Bower R.S., Nwojo M.E., Taussky P., Wetjen N.M., Varzoni T.C., Hanel R.A., Meyer F.B. (2013). Surgical outcomes and predictors of stroke in a north American white and African American moyamoya population. Neurosurgery.

[20] Thines L., Petyt G., Aguettaz P., Bodenant M., Himpens F.-X., Lenci H., Henon H., Gauthier C., Hossein-Foucher C., Cordonnier C. (2015). Surgical management of Moyamoya disease and syndrome: Current concepts and personal experience. Rev. Neurol..

[21] Tho-Calvi S.C., Thompson D., Saunders D., Agrawal S., Basu A., Chitre M., Chow G., Gibbon F., Hart A., Tallur K.K. (2018). Clinical features, course, and outcomes of a UK cohort of pediatric moyamoya. Neurology.

[22] Porras J.L., Yang W., Xu R., Garzon-Muvdi T., Caplan J.M., Colby G.P., Coon A.L., Ahn E.S., Tamargo R.J., Huang J. (2018). Effectiveness of Ipsilateral Stroke Prevention Between Conservative Management and Indirect Revascularization for Moyamoya Disease in a North American Cohort. World Neurosurg..

[23] Deng X., Gao F., Zhang D., Zhang Y., Wang R., Wang S., Cao Y., Zhao Y., Pan Y., Ye X. (2018). Effects of different surgical modalities on the clinical outcome of patients with moyamoya disease: A prospective cohort study. J. Neurosurg..

[24] Arai K., Lok J., Guo S., Hayakawa K., Xing C., Lo E.H. (2011). Cellular mechanisms of neurovascular damage and repair after stroke. J. Child Neurol..

[25] Fung L.-W.E., Thompson D., Ganesan V. (2005). Revascularisation surgery for paediatric moyamoya: A review of the literature. Childs Nerv. Syst..

[26] Kraemer M., Karakaya R., Matsushige T., Graf J., Albrecht P., Hartung H.-P., Berlit P., Laumer R., Diesner F. (2018). Efficacy of STA-MCA bypass surgery in moyamoya angiopathy: Long-term follow-up of the Caucasian Krupp Hospital cohort with 81 procedures. J. Neurol..

[27] Hallemeier C.L., Rich K.M., Grubb R.L., Chicoine M.R., Moran C.J., Cross D.T., Zipfel G.J., Dacey R.G., Derdeyn C.P. (2006). Clinical features and outcome in North American adults with moyamoya phenomenon. Stroke.

[28] Chiu D., Shedden P., Bratina P., Grotta J.C. (1998). Clinical features of moyamoya disease in the United States. Stroke.

[29] Mesiwala A.H., Sviri G., Fatemi N., Britz G.W., Newell D.W. (2008). Long-term outcome of superficial temporal artery-middle cerebral artery bypass for patients with moyamoya disease in the US. Neurosurg. Focus.

[30] Lee J.Y., Phi J.H., Wang K.-C., Cho B.-K., Shin M.-S., Kim S.-K. (2011). Neurocognitive profiles of children with moyamoya disease before and after surgical intervention. Cerebrovasc. Dis..

