# Supplementary material for: Patients with Moyamoya Vasculopathy Evaluated at a Single-Center in The Netherlands; Clinical Presentation and Outcome
Source: J Clin Med. 2021 Apr 27;10(9):1898. doi: 10.3390/jcm10091898 (PMC8124614; doi:10.3390/jcm10091898)
Supplement: Supplementary file 1 [file jcm-10-01898-s001.zip › jcm-1117387-supplementary.pdf]

SUPPLEMENTARY MATERIALS.

Patients with moyamoya vasculopathy evaluated at a single-center in the Netherlands; clinical presentation and outcome.

Table S1. Associated disorders.

Associated disease	Children (n=31)	Adults (n=33)
Fibromuscular dysplasia	10	
Down syndrome	2	
Neurofibromatosis type-1 (NF-I)	1	1
Hereditary spherocytosis	1	
Sickle cell disease	1	
Trisomy 6/17q25 deletion syndrome	1	
NF1/radiotherapy	1	
Atherosclerosis		1
Antiphospholipid syndrome		1
Radiotherapy		1
Sneddon's syndrome		1

Table S2. Ethnicity or country of origin.

Ethnicity *or* country of origin	All			Surgery			Non-surgical		
	All n(%)	Children n(%)	Adults n(%)	All n(%)	Children n(%)	Adults n(%)	All n(%)	Children n(%)	Adults n(%)
Caucasian	42 (65.6)	18 (58.1)	24 (72.7)	28 (66.7)	14 (60.9)	14 (73.7)	14 (63.6)	4 (50)	10 (71.4)
Asian	3 (4.7)	1 (3.2)	2 (6.1)	2 (4.8)	1 (4.3)	1 (5.3)	1 (4.5)	0	1 (7.1)
Northern African	3 (4.7)	2 (6.5)	1 (3)	3 (7.1)	2 (8.7)	1 (5.3)	0	0	0
Somalia	1 (1.6)	1 (3.2)	0	0	0	0	1 (4.5)	1 (12.5)	0
Turkey	1 (1.6)	0	1 (3)	0	0	0	1 (4.5)	0	1 (7.1)
Brazil	1 (1.6)	1 (3.2)	0	1 (2.4)	1 (4.3)	0	0	0	0
Cape Verde	1 (1.6)	0	1 (3)	1 (2.4)	0	1 (5.3)	0	0	0
Aruba	1 (1.6)	0	1 (3)	0	0	0	1 (4.5)	0	1 (7.1)
Iran	1 (1.6)	1 (3.2)	0	1 (2.4)	1 (4.3)	0	0	0	0
Unknown	3 (4.7)	3 (9.7)	0	1 (2.4)	1 (4.3)	0	2 (9.1)	2 (25)	0
Parent/ancestors	7 (10.9)	4 (12.9)	3 (9.1)	5 (11.9)	3 (13)	2 (10.5)	2 (9.1)	1 (12.5)	1 (7.1)
Indonesian	4 (6.3)	2 (6.5)	2 (6.1)	4 (9.5)	2 (8.7)	2 (10.5)	0	0	0
Spanish	1 (1.6)	1 (3.2)	0	1 (2.4)	1 (4.3)	0	0	0	0
Southern African	1 (1.6)	1 (3.2)	0	0	0	0	1 (4.5)	1 (12.5)	0
Asian	1 (1.6)	0	1 (3)	0	0	0	1 (4.5)	0	1 (7.1)

Table S3 Modified Rankin scale at follow-up.

mRS	Improved	Stable	Deteriorated
All	18 (31)†	23 (40)	17 (29)
Childhood-onset*	8 (32)	11 (44)	6 (24)
Adult-onset	10 (30)	12 (36)	11 (33)
Surgical	11 (30)	16 (43)	10 (27)
Childhood-onset*	6 (33)	8 (45)	4 (22)
Adult-onset	5 (26)	8 (42)	6 (32)
Non-surgical	7 (33)	7 (33)	7 (33)
Childhood-onset*	2 (29)	3 (42)	2 (29)
Adult-onset	5 (36)	4 (28)	5 (36)

mRS= modified Rankin Scale.
*9 patients had childhood-onset of MMV and were adults at follow-up; †of 6 children (5 surgical and 1 non-surgical) no baseline mRS was available (<age 4).

Table S4. Univariable logistic regression analysis.

Predictors	**poor outcome (mRS ≥3)**		**Recurrent stroke**		**No normal school/ adult does not work**	
	Odds ratio (95% CI)	p value	Odds ratio (95% CI)	p value	Odds ratio (95% CI)	p value
Age of onset MMV	1.010 (0.977-1.043)	0.574	1.017 (0.984-1.052)	0.311	1.013 (0.980-1.047)	0.447
Diagnosis (MMD or MMS)	1.007 (0.272-3.726)	0.992	1.713 (0.480-6.114)	0.407	2.152 (0.578-8.015)	0.253
mRS presentation ≥3	**3.828 (1.073-13.664)**	**0.039**	1.455 (0.407-5.197)	0.564	2.632 (0.704-9.838)	0.150
# events (ICH/infarction) before presentation NSC	1.021 (0.583-1.788)	0.943	0.766 (0.412-1.424)	0.400	1.436 (0.795-2.596)	0.231
Surgically treated	**0.240 (0.075-0.771)**	**0.017**	*0.314 (0.099-0.998)*	*0.050*	0.978 (0.319-2.994)	0.969

CI= confidence interval; ICH: intracerebral hematoma; MMD=moyamoya disease; MMS=moyamoya syndrome; MMV= moyamoya vasculopathy; mRS= modified Rankin Scale; NSC= neurosurgical center; #=number.
